# Vitamin D status and cardiometabolic risk factors among hypertensive and normotensive adults: a hospital-based cross-sectional study in Nepal

**DOI:** 10.1186/s40795-026-01247-x

**Published:** 2026-01-12

**Authors:** Ojaswee Sherchand, Prabin Adhikari, Krishna Chandra Devkota, Saroj Kunwar

**Affiliations:** 1 Department of Biochemistry, Nepal Medical College and Teaching Hospital, Kathmandu, Nepal; 2 Department of Internal Medicine, Nepal Medical College and Teaching Hospital, Kathmandu, Nepal

**Keywords:** Vitamin D deficiency, Hypertension, Dyslipidemia, Abdominal obesity, Family history, South Asia, Nepal

## Abstract

**Background:**

Vitamin D deficiency is increasingly recognised as a risk factor for non-communicable diseases, including hypertension. However, evidence on their relationship remains inconsistent, particularly in middle and low-income countries like Nepal. This study aimed to assess the prevalence of vitamin D deficiency among hypertensive and normotensive adults, and identify the demographic, anthropometric and clinical risk factors among hypertensive adults attending a tertiary care hospital in Kathmandu, Nepal.

**Methods:**

A hospital-based cross-sectional study was conducted among 108 adults (54 hypertensive and 54 normotensive) at Nepal Medical College and Teaching Hospital from September 2024 to March 2025. Data on sociodemographic factors, anthropometric variables and clinical parameters were collected. Serum 25(OH)D was measured using Chemiluminescence Immunoassay (CLIA), and was categorised as sufficient (≥ 30 ng/ml), insufficient (20–29 ng/ml) and deficient (< 20 ng/ml). Blood pressure was classified according to the 7th Joint National Committee on the Prevention, Detection, Evaluation and Treatment of High Blood Pressure. Group comparisons were performed using the Chi-Square test, and significant variables were subjected to multiple logistic regression.

**Results:**

Overall, 42.6% were vitamin D deficient. Although deficiency was more prevalent in hypertensive than normotensive, the association was only borderline significant (*p* = 0.05) and did not remain significant after post-hoc correction. In multiple logistic regression, abdominal obesity (AOR: 2.8; 95% CI: 1.2–6.7; *p* = 0.019), dyslipidemia (AOR: 2.8; 95% CI: 1.2–7.2; *p* = 0.024) and having a family history of hypertension (AOR: 2.7; 95% CI: 1.1–6.8; *p* = 0.028) were independently associated with increased odds of hypertension. The overall model explained 24% of variance in hypertensive status (Nagelkerke R^2^ = 0.24).

**Conclusions:**

Dyslipidemia, abdominal obesity and family history were significant predictors of hypertension in this cohort, while vitamin D deficiency showed a non-significant association with hypertension. Nonetheless, the high prevalence of vitamin D deficiency highlights the need for increased public health awareness and preventive healthcare strategies. Larger population-based studies are needed to understand the role of vitamin D in the prevention and control of hypertension in Nepal.

## Introduction

The World Health Organisation (WHO) states 1.28 billion people are affected by hypertension worldwide, with the highest burden in low and middle-income countries (LMICs) [1]. In Nepal, the WHO STEPwise approach to surveillance (STEPS) conducted in 2019 estimated that the prevalence of hypertension among adults was 24.5%. [2]

The aetiology of hypertension is multifactorial, encompassing both modifiable and non-modifiable factors [3]. Recently, hypovitaminosis D has been proposed as a modifiable risk factor for hypertension due to its role in regulating the renin angiotensin aldosterone system and vascular smooth muscle function [4]. However, findings across international studies are inconsistent, with some reporting an inverse relationship between serum 25(OH)D levels and hypertension, while others find no significant relationships [5–7].

Despite increasing interest, there is limited evidence from South Asian countries, particularly Nepal, regarding the potential role of vitamin D deficiency in hypertension. The cultural and dietary habits, limited vitamin D-fortified foods, and reduced sunlight exposure due to urbanisation create a distinct risk profile for Nepal, which needs to be studied [8].

This study aims to (1) assess the prevalence of vitamin D deficiency among hypertensive and normotensive adults, and (2) identify the demographic, anthropometric and clinical risk factors among hypertensive and normotensive adults attending a tertiary care hospital in Kathmandu, Nepal. To our knowledge, this is the first hospital-based cross-sectional study in Nepal that directly compares vitamin D status between hypertensive and normotensive adults in a tertiary care setting in Nepal.

## Materials and methods

### Study design and setting

This hospital-based comparative study was conducted at Nepal Medical College and Teaching Hospital (NMCTH) in Gokarneshwar Municipality, Kathmandu, Nepal, from September 2024 to March 2025. NMCTH was selected as it is a major tertiary care institute serving a diverse catchment population in urban and peri-urban Kathmandu. The study received ethical approval from the Institutional Research Committee (Ref. No.: l7-081/082), and written informed consent was obtained from all participants.

### Participant recruitment

Participants were recruited using consecutive sampling from the patients visiting the Biochemistry laboratory of NMCTH during the study period. Hypertensive cases were identified as clinician-confirmed diagnoses of hypertension based on JNC 7 criteria (systolic blood pressure readings ≥ 140 mmHg and/or diastolic pressure readings ≥ 90 mmHg) and/or current use of antihypertensive medication [9]. Normotensive controls were recruited from individuals visiting the same laboratory who reported no prior diagnosis of hypertension and had a normal blood pressure reading on measurement.

### Inclusion and exclusion criteria

Inclusion Criteria:

- Adults 18 to 80 years
- Providing written consent to participate in the study

Exclusion Criteria:

- Pregnant and lactating women
- those with renal failure, chronic inflammatory diseases, or cancer
- Patients using vitamin D supplements in the last six months

### Sample size calculation

The sample size was calculated using the following formula:

$$\mathrm N1=\frac{\;(\mathrm\sigma1^2+\mathrm\sigma2^2/\mathrm\kappa)\;{(\mathrm Z_{1-\mathrm\alpha/2}+\mathrm Z_{1-\mathrm\beta})}^2}{\triangle^2}$$

where;

σ_1_ = standard deviation of group 1 control (12.2) [10].

σ_2_ = standard deviation of group 2 cases (19.4).

κ = n_1_/n_2_ = 100/100 = 1.

Z_1-α/2_ = 2 sided Z value (Z = 1.96 for 95% confidence interval) = 1.96.

Z_1-β_ = power = 0.84.

**∆ = **difference between the mean scores = 15.3- 24.5 = 9.2

$$\mathrm{N}=\frac{\;\left(148.84\;+\;376.36/1\right)\left(1.96\;+\;0.84\right)}{84.64}$$

 = 49 cases and control each; Total sample = 98.

Ten percent was added for non-respondents resulting in 108 total sample.

### Data collection and measurements

Sociodemographic and clinical data were collected using a structured questionnaire. Physical Activities were assessed using the short format of the International Physical Activity Questionnaires (IPAQ) and was classified metabolic equivalents (MET) minutes per week of activity as < 600 MET: physically inactive, ≥ 600 MET: physically active [11].

#### Anthropometric measurements

Weight and height were measured by trained nurses using standardized protocols with participants in light clothing and no shoes. BMI was calculated as kg/m^2^. BMI was categorized according to the WHO classification for Asians as normal weight (18.5–22.9 kg/m^2^), overweight (≥ 23.0–24.99 kg/m^2^), and obese (≥ 25 kg/m^2^) [12, 13]. Waist circumference (for abdominal obesity), was measured at the midpoint between the lower margin of the last rib and the iliac crest using a non-stretchable tape; two measurements were taken and averaged. Waist circumference ≥ 90 cm in men and ≥ 80 cm in women were categorized as having central obesity [14].

#### Blood pressure measurement

Blood pressure was measured using a calibrated aneroid sphygmomanometer. Two readings were taken 5 min apart with the participants seated, and the average was used. Hypertension was defined as systolic blood pressure readings ≥ 140 mm Hg and/or diastolic pressure readings ≥ 90 mm Hg and or current use of antihypertensive medication from JNC 7 criteria [9]. Although newer classifications (JNC 8, ACC/AHA 2017) exist, JNC 7 was chosen to ensure comparability with Nepalese clinical practice and previous local studies.

Dyslipidemia was defined according to the National Cholesterol Education Program Adult Treatment Panel III (NCEP-ATP III) criteria: total cholesterol ≥ 200 mg/dl, LDL-C ≥ 130 mg/dl, triglyceride ≥ 150 mg/dl or HDL-C < 40 mg/dl in men and < 50 mg/dl in women or by self-reported use of lipid-lowering medications (e.g. statins) [15].

#### Serum vitamin D estimation

Venous blood samples were collected under aseptic precautions in the morning and fasting was not mandatory for vitamin D measurement. Serum vitamin D 25(OH) was assessed using the Chemiluminescence immunoassay (CLIA) in the Beckman Access 2 analyzer in the Department of Biochemistry. Vitamin D was categorized as: ≥ 30 ng/ml is considered sufficient, 20–29 ng/ml as insufficient and < 20 as deficient [16]. To ensure the accuracy, dependability, and quality of the tests, the internal quality control established by the manufacturer was applied.

### Statistical analysis

Statistical analysis was done using Statistical Package for Social Science (SPSS) version 20. Categorical data were expressed as numbers (percentage) n (%). The association between the variables in the hypertensive and normotensive groups was compared using Pearson’s Chi-square test. Crude odds ratio (OR) and 95% Confidence Interval (CI) were calculated to estimate the strength of association in bivariate analysis. A *p*-value of less than 0.05 was regarded as statistically significant. For categorical variables with more than two categories that showed borderline or significant associations (e.g. vitamin D), Bonferroni-adjusted post-hoc pair-wise comparisons were conducted to identify differences between the categories. Variables found to have significant associations in Chi-Square tests were further subjected to multiple logistic regression with hypertension as binary outcome. Adjusted odds ratio (AOR) and 95% CI were reported, and model fit was assessed with Nagelkerke R^2^.

## Results

### Participant characteristics

A total of 108 participants were enrolled, including 54 hypertensive and 54 normotensive adults. The mean age of the participants was higher in the hypertensive group, with participants aged ≥ 50 years having significantly higher odds of hypertension than normotensives (OR:0.4, CI:0.2–0.9, *p* = 0.03). Women comprised 58% of the sample but gender was not significantly associated with hypertension (OR:0.7, 95% CI:0.3–1.7, *p* = 0.5). The ethnic distribution included Khas (35.2%), Adivasi/Janajati (29.6%) and other ethnicities (35.2%), no significant difference in hypertension prevalence was observed across ethnic groups (*p* = 0.7). 50.9% participants were residents of Gokarneshwor Municipality, but residence was not associated with hypertension (OR:1.4, 95% CI:0.6–3.1, p = 0.3). Other sociodemographic variables including gender, marital status, occupation, smoking, alcohol consumption, showed no significant difference between the groups (Table 1).

**Table 1:** Sociodemographic variables of the study population

Variables	Hypertensive	Normotensive	Total n (%)^a^	*P*-value	OR (95%CI)^b^
Ethnicity					
Khas	18(16.7)	20(18.5)	38(35.2)	0.7	1(0.3–2.6)
Other ethnicities	21(19.4)	17(15.7)	38(35.2)		1.4(0.5–3.5)
Adivasi/Janajati	15(13.9)	17(15.7)	32(29.6)		Reference
Gender					
Male	21(19.4)	24(22.2)	45(41.7)	0.5	0.7(0.3–1.7)
Female	33(30.6)	30(27.8)	63(58.3)		Reference
Residential Municipality					
Gokarneshwor	30(27.8)	25(23.1)	55(50.9)	0.3	1.4(0.6–3.1)
Outside Gokarneshwor	24(22.2)	29(26.9)	53(49.1)		Reference
Marital Status					
Married	41(38)	45(41.7)	86(79.6)	0.3	0.6(0.2–1.6)
Others^c^	13(12)	9(8.3)	22(20.4)		Reference
Age Group					
< 50 years	20(18.5)	31(28.7)	51(47.2)	0.03	0.4(0.2–0.9)
≥ 50 years	34(31.5)	23(21.3)	57(52.8)		Reference
Diet					
Non-vegetarian	46(42.6)	45(41.7)	91(84.3)	0.7	1.1(0.4–3.2)
Vegetarian	8(7.4)	9(8.3)	17(15.7)		Reference
Smoking					
Yes	12(11.1)	9(8.3)	21(19.4%)	0.4	1.4(0.5–3.7)
No	42(38.9)	45(41.7)	87(80.6%)		Reference
Alcohol					
Yes	18(16.7%)	23(21.3%)	41(38%)	0.3	0.6(0.3–1.4)
No	36(33.3%)	31(28.7%)	67(62%)		Reference
Occupation					
Earning a living	28(25.9)	30(27.8)	58(53.7)	0.7	0.8(0.4–1.8)
Unemployed	26(24.1)	24(22.2)	50(46.3)		Reference

*P* values < 0.05 are significant

^a^Total n (%) represents the number and percentage of rows. Comparison of parameters between the groups control was performed using χ2 test

^b^OR(95%CI) is odds ratio with 95% confidence interval

^c^Others: unmarried, divorced, widowed

### Vitamin D Status

**Fig. 1:**
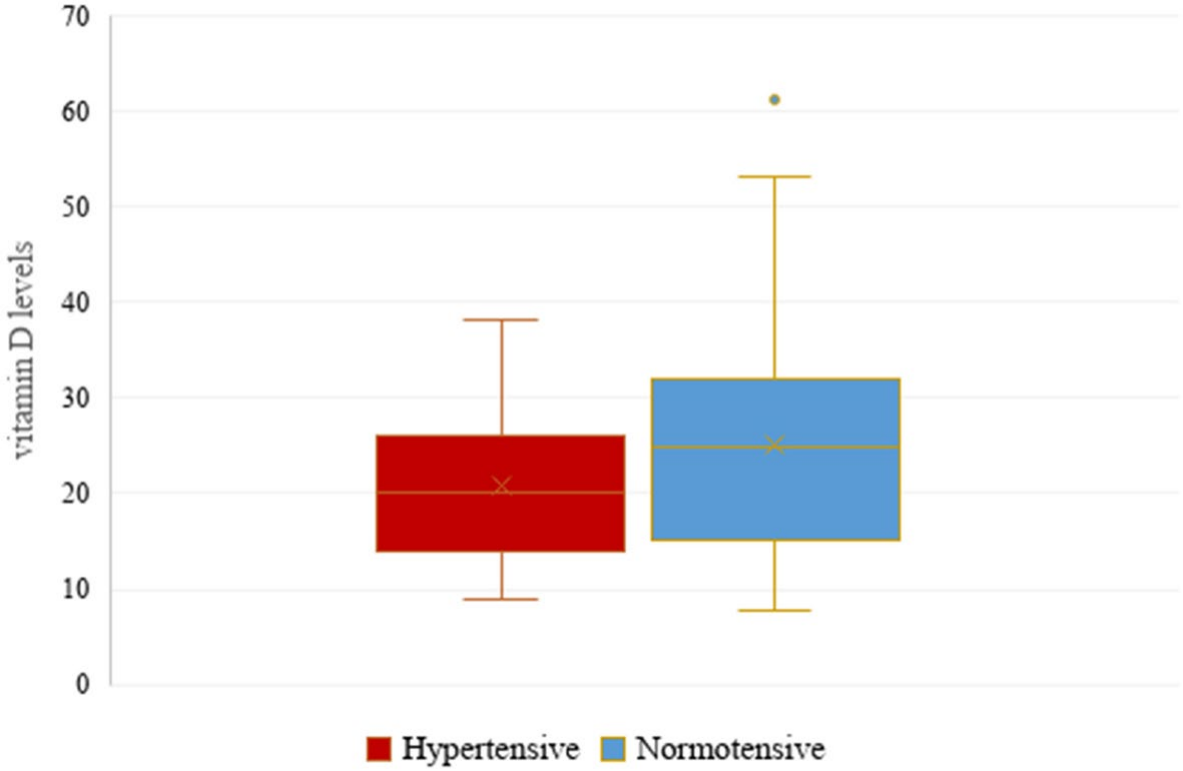
Box-and-whisker plots showing vitamin D levels between the two groups. Box-and-whisker plots showing vitamin D levels between the two groups. Each boxplot shows the median, the 25th and 75th percentiles (lower and upper hinges), and the 5th and 95th percentiles (whiskers)

The median and 25th and 75th percentiles of serum vitamin D levels in hypertensives and normotensives were 20(14,25) and 24.9(15.3,32), respectively (Fig. 1). The overall prevalence of Vitamin D deficiency was 42.6%, while 34.2% had vitamin D insufficiency. Vitamin D deficient individuals had higher odds of hypertensive compared to those with sufficient vitamin D (OR:3.4, 95%CI: (1.1–9.9, p = 0.051). Vitamin D Insufficient category also showed greater odds of hypertension, though this was not statistically significant (OR:2.6, 95%CI:0.9–7.9). The overall association of vitamin D status and hypertension was borderline significant (χ2 p-value = 0.05). However, Bonferroni-adjusted post-hoc test revealed no significant pairwise differences between deficienct, insufficient and sufficient categories (all p > 0.017). (Table 2).

**Table 2:** Vitamin D Levels among Hypertensive and Normotensives

Vitamin D status	Hypertensive	Normotensive	Total n (%)	*P*-value	OR (95%CI)
Deficiency	27(25)	19(17.6)	46(42.6)	0.05^a^	3.4(1.1–9.9)
Insufficiency	20(18.5)	18(16.7)	38(35.2)		2.6(0.9–7.9)
Sufficient	7(6.5)	17(15.7)	24(22.2)		Reference

Total n(%) represents the number and percentage of rows. Vitamin D levels of ≥ 30 ng/ml considered sufficient, 20–29 ng/ml as insufficient and < 20 as deficient. Comparison of parameters between the groups was performed using the Chi Square test

^a^Due to borderline p-value in *χ*^*2*^ test, bonferroni post-hoc test was done which revealed no statistical significance, *p* > 0.017

### Clinical and anthropometric variables

Abdominal Obesity was present in 35.2% hypertensive participants compared to 24.1% normotensive and was significantly associated with increased odds of hypertension (OR:2.5, 95%CI: 1.2–5.6, *p* = 0.01). Dyslipidemia was observed in 23.2% of hypertensive participants vs 10.2% normotensives, with affected individuals having three times higher odds of hypertension (OR:3.3, 95%CI:1.4–7.8, *p* = 0.004). A positive family history of hypertension was also associated with increased odds of hypertension (OR: 2.4, 95%CI:1.1–5.5, *p* = 0.02). In contrast, BMI, smoking, alcohol consumption, physical activities and comorbidities did not show a significant association with hypertension.(Table 3).

**Table 3:** Different covariates among Hypertensive and Normotensive

Variables	Hypertensive	Normotensive	Total n (%)	*P*-value	OR (95%CI)
Co-morbidities^a^					
Present	28(25.9)	27(25)	55(50.9)	0.8	1(0.5–2.2)
None	26(24.1)	27(25)	53(49.1)		Reference
BMI					
Obese	37(34.3)	36(33.3)	73(67.6)	0.8	1(0.4–2.4)
Not obese	17(15.7)	18(16.7)	35(32.4)		Reference
BMI mean (SD)	26.6 (4.4)	25.5 (3.7)			
Waist circumference					
Abdominal obesity present	38(35.2)	26(24.1)	64(59.3)	0.01	2.5(1.2–5.6)
No obesity	16(14.8)	28(25.9)	44(40.7)		Reference
Waist mean (SD)	36.3(4.8)	34.6(4.6)			
Dyslipidemia					
Present	25(23.1)	11(10.2)	36(33.3)	0.004	3.3(1.4–7.8)
Absent	29(26.9)	43(39.8)	72(66.7)		Reference
Physical activities					
Below 600MET	22(20.4)	23(21.3)	45(41.7)	0.8	0.9(0.4–1.9)
≥ 600 MET	32(29.6)	31(28.7)	63(58.3)		Reference
Family history of HTN					
present	25(23.1)	14(13)	39(36.1)	0.02	2.4(1.1–5.5)
No	29(26.9)	40(37)	69(63.9)		Reference

Total n (%) represents the number and percentage of rows. Comparison of parameters between hypertensive and normotensive was performed using χ2 test

*P* values < 0.05 are significant

^a^Comorbidities includes diabetic, thyroid disorders, nerve disorders

### Multiple logistic regression

In multiple logistic regression model including age group, dyslipidemia, abdominal obesity, family history of hypertension, three factors were independently associated with hypertension (Table 4). Participants with dyslipidemia had nearly three times higher odds of hypertension compared to those without dyslipidemia (AOR: 2.8; 95% CI: 1.2–7.2; p = 0.024). Abdominal obesity was also significantly associated with increased odds of hypertension (AOR: 2.8; 95% CI: 1.2–6.7; p = 0.019). Similarly, individuals with a positive family history of hypertension had close to three times higher odds of being hypertensive (AOR: 2.7; 95% CI: 1.1–6.8; p = 0.028). Older age ≥ 50 years was not found to have a significant association with hypertension after adjusting for confounding variables (p = 0.94). The overall model explained 24% of variances in hypertension status (Nagelkerke R^2^: 0.24).

**Table 4:** Multiple logistic regression showing crude and adjusted Odds Ratio for Hypertension

**Variables**		**Crude OR (95% CI)**	***p*-value**	**Adjusted OR (95% CI)**	***p*-value**
Age group	≥ 50 years	2.3(1–4.9)	0.35	2(0.8–4.8)	.094
Dyslipidemia	Present	3.3 (1.3–8)	**0.002**	2.8(1.2–7.2)	**.024**
Abdominal Obesity	Present	2.5(1.2–5.6)	**0.01**	2.8(1.2–6.7)	**.019**
Family history	Present	2.5(1.1–5.5)	**0.02**	2.7(1.1–6.8)	**.028**

*OR* Odds ratio, *CI* Confidence interval, *Adjusted OR* Adjusted Odds Ratio, adjusted model includes age group, dyslipidemia, abdominal obesity and Family history. Statistically Significant *p*-values < 0.005 shown in bold. Nagelkerke square for the model is 0.24

## Discussion

The prevalence of vitamin D deficiency was 42.6% in our study. While vitamin D deficiency was common, it was not independently associated with hypertension. Instead, abdominal obesity, dyslipidemia, and positive family history emerged as strong predictors consistent with evidence from other South Asian countries [17, 18]. In the Nepalese context, these associations are particularly relevant due to the high carbohydrate-based diet, limited physical activity in urban populations, and increasing prevalence of metabolic syndrome. Importantly, given the hospital-based setting, we caution against overgeneralizing our findings to the wider community population.

Abdominal obesity is believed to be associated with increased visceral fat accumulation, which is connected to heightened insulin resistance contributing to atherosclerosis and hypertension [19]. The National Family Health Survey 2019–21 (NFHS‐5) in India reported abdominal obesity as a significant risk factor for hypertension in adults, while data from Bangladesh showed associations between dyslipidemia and hypertension [20, 21].

Family history also emerged as a significant determinant. Familial studies show that genetic factors account for 30% of blood pressure variability, with values ranging from 25% in pedigree studies to 65% in twin studies [22]. These findings may reflect both genetic predisposition and shared lifestyle or environmental exposures within families. Given that this is a non-modifiable risk factor, it highlights the need for targeting screening and preventive strategies in individuals with a known family history of hypertension.

Interestingly, while this study aimed to explore the potential link between vitamin D deficiency and hypertension, vitamin D status did not show a significant association with hypertension after Bonferroni correction. This contrasts with prior studies suggesting an inverse relationship between serum 25(OH)D with blood pressure but supports other recent meta-analyses and randomized trials that found limited evidence for causality [5, 10]. A retrospective study conducted in California, United States, found significantly higher incidences of hypertension amongst patients with vitamin D below 40 ng/ml [23]. This suggests that perhaps the optimal level of vitamin D for different health aspects as blood pressure, varies from the universally accepted > 30 ng⁄mL. The small sample size of the study did not allow us to test this theory. Further studies with a longitudinal design and larger sample sizes may help clarify the association in South Asian context.

### Strengths and limitations

The strength of this study includes the use of objectively measured biochemical and anthropometric data and the analysis of multiple relevant risk factors. However, the cross-sectional design limits causal inference. The hospital-based design limits generalisation to the wider community, potentially limiting external validity. The samples were collected from September to March, spanning both autumn and winter seasons, which we acknowledge may influence vitamin D levels; however, seasonality or sunlight exposure data were not adjusted for in the analysis. Also, residual confounders and self-reported variables as family history, may have introduced bias. Other biochemical markers that could potentially influence the vitamin D level were not measured. Future research incorporating comprehensive biochemical assessments could provide deeper insights into the relationship between vitamin D status and hypertension in the Nepalese population.

## Conclusion

In this study, dyslipidemia, abdominal obesity and family history of hypertension were significantly associated with increased odds of hypertension. These findings highlight the need for integrated screening and management strategies focusing on metabolic health in both hypertensive and normotensive individuals. However, it is important to acknowledge that our findings are from a hospital-based setting and may not fully reflect the general population. Hence, this study warrants the need to conduct larger population-based studies to validate its associations and guide national-level preventive strategies.

## Data Availability

The unprocessed data and other raw data supporting the conclusions of the study will be made available from the corresponding authors upon reasonable request [drojasweesherchand@hotmail.com](mailto:drojasweesherchand@hotmail.com).

## Abbreviations

25(OH)D: 25-Hydroxy vitamin D
BMI: Body mass Index
CLIA: Chemiluminescence Immunoassay
MET: Minutes per week
HTN: Hypertension
IPAQ: International Physical Activity Questionnaires
LMICs: Low and middle-income countries
JNC: Joint National Committee on Prevention, Detection, Evaluation, and Treatment of High Blood Pressure
NMCTH: Nepal Medical College and Teaching Hospital
VDR: Vitamin D receptor
