# Correction: Vitamin D status and cardiometabolic risk factors among hypertensive and normotensive adults: a hospital-based cross-sectional study in Nepal

**DOI:** 10.1186/s40795-026-01348-7

**Published:** 2026-05-20

**Authors:** Ojaswee Sherchand, Prabin Adhikari, Krishna Chandra Devkota, Saroj Kunwar

**Affiliations:** 1 Department of Biochemistry, Nepal Medical College and Teaching Hospital, Kathmandu, Nepal; 2 Department of Internal Medicine, Nepal Medical College and Teaching Hospital, Kathmandu, Nepal

**Correction: BMC Nutr 12**,** 28 (2026)**

**https://doi.org/10.1186/s40795-026-01247-x**

Following the publication of the original article [1], the author’s name Krishna Chandra Devkota was incorrectly written as Chandra Krishna Devkota.

This has been corrected above and the original article [1] has been updated.
